# Risk Perceptions on Hurricanes: Evidence from the U.S. Stock Market

**DOI:** 10.3390/ijerph14060600

**Published:** 2017-06-05

**Authors:** José Manuel Feria-Domínguez, Pilar Paneque, María Gil-Hurtado

**Affiliations:** 1 Department of Financial Economics, Pablo de Olavide University, 41013 Seville, Spain; 2 Department of Geography, Pablo de Olavide University, 41013 Seville, Spain; ppansal@upo.es; 3 EY Climate Change and Sustainability Assurance, 41013 Seville, Spain; maria.del.carmen.gil.hurtado@es.ey.com

**Keywords:** natural hazards, hurricanes, risk perceptions, financial markets, event study

## Abstract

This article examines the market reaction of the main Property and Casualty (P & C) insurance companies listed in the New York Stock Exchange (NYSE) to seven most recent hurricanes that hit the East Coast of the United States from 2005 to 2012. For this purpose, we run a standard short horizon event study in order to test the existence of abnormal returns around the landfalls. P & C companies are one of the most affected sectors by such events because of the huge losses to rebuild, help and compensate the inhabitants of the affected areas. From the financial investors’ perception, this kind of events implies severe losses, which could influence the expected returns. Our research highlights the existence of significant cumulative abnormal returns around the landfall event window in most of the hurricanes analyzed, except for the Katrina and Sandy Hurricanes.

## 1. Introduction 

In the context of climate change, natural disasters have emerged as a relevant topic in the recent academic literature, being addressed from a multidisciplinary point of view. One of the most harmful disasters is the hurricane. According to the National Aeronautics and Space Administration (NASA) (2012), hurricanes are defined as large, rotating thunderstorms, which form over tropical and subtropical bodies of water. They usually take place in the tropical latitudes and most of them appear at the end of the summer, as it is the time when the temperature of the seawater is higher than during the rest of the year. Hurricanes are classified by the Saffir-Simpson Hurricane Wind Scale (SSHWS) into five main categories, being the category one hurricanes with the lowest wind speed and the category five the highest one. It is considered that, once a storm reaches the first category, this turns into a hurricane. The minimum gust of wind required considering a storm as a hurricane of category one is 119 km/h. The tropical storm activity in the Atlantic Basin has increased over the past decade and a half, and the effect of powerful storms, such as hurricanes, on assets prices in several important markets has been addressed in the financial literature [1]. 

As Horwich [2] states, natural disasters are localized events and they may affect a limited part of the whole economy. These catastrophic events, among others [3], play an important role in the value of companies, in special in those related to Property and Casualty Insurance companies (P & C (Property and Casualty) Companies). This field is extremely fragile as the external environment influences and affects these companies. In 2013, hurricanes represented 42% of all United States (U.S.) insured catastrophe losses. The U.S. insurance market is the largest in the world, amounting 34% of the global non-life insurance premiums (AXCO, London, UK, 2013). A hurricane can produce the interruption of petrol production and even produce a spill over after it. It can also produce a decrease in the number of exports due to the interruption of the production of several industries, a decrease in the tourism and trades, decrease in the labor market, etc. The losses derived from hurricanes accounts for billions of dollars [4]. Hurricanes have immediate effects on an economy and these effects may persist for some time in the near future [5]. Surprisingly, very little research has examined the effect of hurricanes on insurance stock prices. Lamb [6] and Angbazo and Narayanan [7] focused exclusively on property and liability firms and examined how a hurricane affected insurer stock prices using event study methodology, but did not take the storm characteristics into account in the analysis. Rather, they focused only on the timing of hurricane landfall. According to the NHC, landfall is defined as “*the intersection of the surface centre of a tropical cyclone with a coastline*”. Since it is possible for a cyclone to produce the highest wind speed over water or even once the landfall occurs [5], we have also considered some characteristics such as the wind speed, the geographical area or the time of the storm. All of them are key variables for the developing of a hurricane and play an important role in the final damage produced.

The way in which a hurricane can influence the stock market is a subject to be studied, as there is not a unanimous response. It is assured there is a relationship among natural disasters, stock markets and their performance. It is known that the industry is damaged; for instance, when there are power outages for several days or even weeks that isolate areas and population and high-speed winds pull down light poles. Both electricity and petroleum industries are affected by these events and this is consequently shown in the stock markets.

On the other hand, a positive effect may arise from hurricanes: improvement in productivity due to the reconstruction that implies a hurricane. In addition, a positive effect is hypothesized due to payments on claims and a positive effect may be due to expectations of higher future premiums [5]. The reconstruction that implies a hurricane increases the productivity of the economy. This is an opportunity for re-investment and replacement of capital goods, which can positively affect growth [8]. On balance, this double effect produces a serious damage on industrial capacity and on some industries but a positive effect arising from productivity and reconstruction takes place even being able to produce a reduction in the unemployment rate.

Another type of reaction apart from positive or negative can be found, a neutral reaction in which the situation “ceteris paribus”. Lamb [6] argues that the market may discriminate among firms in relation to their geographic risk exposure. To clarify this point, we carry out a standard short-term event study to calibrate the reaction of the main U.S. P & C Companies, quoted in the U.S. stock market, to some recent hurricanes.

The remainder of this paper is organized as follows: Section 2 describes the sample and data used; Section 3 provides a theoretical background of the event study methodology; Section 4 explains the design of our research; Section 5 presents the main findings and results; and Section 6 presents the conclusions.

## 2. Data and Sample

To conduct our analysis, we have selected two sets of data. The first set corresponds to seven hurricanes that hit an area of the U.S. Atlantic Coast during 2005–2012. The second selection is the main P & C Insurance Companies listed on the New York Stock Exchange (NYSE): Marsh & McLennan Companies (MMC), Progressive Corporation (PGR), Ace Limited (ACE), The Allstate Corporation (ALL), The Chubb Corporation (CB), Travellers (TRV) and Berkshire Hathaway’s (BRK-B). Table 1 summarizes the sample of the hurricanes selected, ranked by the Saffir-Simpsom Hurricane Wind Scale (SSHWS). 

Most of these data have been gathered from the National Hurricane Centre (NHC) as they are unbiased and publicly available (see more at: http://www.nhc.noaa.gov).

On the other hand, Table 2 corresponds to the main P & C Companies, listed in the NYSE, whose annual revenues in 2013 are greater than $10,000 million. All of them were established a long time ago, ensuring that the impact of the hurricanes could be widely measured on terms of stock returns.

The initial sample is composed of seven recent hurricanes. They were chosen according to some parameters. In a concern of sufficient impact, all the hurricanes selected reached at least the third category or higher and hit the same area of the U.S. during the hurricane season, from 1 June to 30 November, corresponding to 2005 to 2012. Furthermore, all of them produced extreme damage on the area and could have produced any abnormal reaction in the stock market. The Hurricane season usually shows a peak from mid-August to late October. However, according to National Oceanic and Atmospheric Administration (NOAA), they can occur anytime in the hurricane season.

Firstly, a pickup was made to know which one of the hurricanes should be studied. All of them have taken place recently, from 2005 to 2012. They were classified depending on their wind speed (specified as Categories 3, 4 and 5). Five categories are found to classify them (Table 3).

Hurricanes have been chosen according to their categories, being the ones with the highest category chosen as they are considered to have a more significant impact on the area. Moreover, some of them, such as Katrina, are the most damaging ever occurred.

For our analysis, we focus on the Atlantic basin as Table 4 reflects. Many regions have suffered hurricanes more than once and sometimes these areas affected are not totally recovered when they receive again the impact of a new hurricane destroying all the reconstruction.

In general, hurricanes are in accordance with the global distribution of natural catastrophes. During 2010, most catastrophes occurred in South America and Asia. Thus, America and Asia suffered from 365 and 310 catastrophes, respectively, followed by Europe with 120, Africa with 90 and Australia with 65. Furthermore, due to the number of catastrophes, North and South America had the largest proportion of insured losses, around 60% of the total loss. From 2000 to 2010, the number of catastrophic events has increased, showing an up-ward tendency, especially during the last three years. This trend suggests that either there has been an increase in the vulnerability to catastrophic events, or that the underlying climatic conditions generating these events now pose a greater risk than they did two decades ago. In addition, the frequency of these catastrophic events varies considerably across different states being Texas the one with the highest rate of annual catastrophic events on average. The result of this tends to be an increase in the risks by the insurers who adapt to these catastrophic risks by raising insurance rates, leading to lower loss ratios after the catastrophic event [9].

However, although many authors argue the number of natural disasters have increased in the last fifty years [10], it does not seem to be the case for hurricanes. Based on the Monthly Atlantic Tropical Weather Summary published by NHC in the last ten years (2004–2014) the number of hurricanes and tropical depressions in the East coast of the U.S. have remain constant without great fluctuations. The average number of these events per year is around 16.73. Most of the years count on numbers far below the average. However, there are some periods with huge fluctuations, e.g., 2005 with 28 hurricanes, producing an increase in this average.

Moreover, the increases of catastrophic events make insurance companies have higher costs in terms of policies and expenses. The majority of the insurance companies do not cover the complete damage, being in some cases less than half of the amount estimated. In practice, it is impossible to forecast the total amount of losses and the total insured losses of any hurricane. All the estimations and figures given are purely hypothetical and theoretical, mainly based on forecasts. Even so, the higher the damage, the more likely it will be reinsurers pay a larger amount for the hurricane. However, in the case of hurricane Ike (2008), the quantity was on average 66% of the reinsurance claims. This has produced many insurer companies to become insolvent or technically insolvent requiring a transfer of funds from others to pay claims. For instance, hurricane Andrew in 1992 made seven domestic insurance companies become insolvent.

Furthermore, in Florida, when an insurance company becomes insolvent, the Florida Insurance Guarantee Association (FIGA) is obliged by law to oversee the claim and pay the amount required to the affected ones. If the money FIGA has in its reserves is not enough for the payment, the association can issue two types of assessments against property and casualty insurance companies, regular assessments and emergency assessments. This was the case of a company in 2004, which became insolvent and an entire group also became insolvent in 2005 due to the hurricane season. Since 2006, a regular assessment is paid to guarantee the proper operability of this association.

Apart from this, new legislations have been created due to numerous claims made recently. Since hurricane Andrew (1992), the insurance companies have reduced progressively their exposures to such events. This sounds a bit ironic, and because of this, they made huge profits just after the hurricane Katrina in 2005 and they have had perfect results during these years despite the current economic situation. In Louisiana, Governor Blanco issued several Executive Orders, with several legal deadlines that were impossible to meet due to the circumstance of damage and devastation occurred in this area. The Louisiana Legislature Enacted Acts 2006, Nos. 739 and 802, “which extend the prescriptive period within which citizens may file certain claims under their insurance policies for losses occasioned by Hurricanes Katrina and Rita”. Before this, “no insurance contract issued in Louisiana could limit the right of action against an insurer to less than twelve months”.

Insurer stock prices should reflect all damages produced by these events. It is supposed the higher the expected damages, the higher the expenditures by insurance companies and this will result in lower earnings and returns for these companies. Datastream is used for obtaining the daily stock closed prices of the P & C Companies as well as the S & P 500 index, for both the estimation window and the event window around the hurricanes landfalls.

## 3. Methodological Background

The objective of our research is to determine whether the effects of these hurricanes influence on the stock prices of the P & C Companies listed at the NYSE. As suggested by Hewitt [11] the event study methodology is the best way to address these kinds of market reactions. Campbell and Wasley [12] point out that it has become common in finance to measure an event’s economic impact by using assets prices observed over a relatively short time period. The type of event is usually motivated by economic theory, for instance how the stock prices behave during and after the ex-dividend day, merger and acquisitions announcements, upgrading and downgrading in ratings, etc. According to Kothari and Warner [13], they have been widely used in the field of law to study the effect of a regulation or to assess damages in legal liability cases. The event study methodology relies on challenging conditions of the Efficient Market Theory [14] to show abnormal returns as a result of this unusual event, in our case, hurricanes. Thus, this influential event, which produces a change in a stock return, leads to the market imputes a charge in the net present value of the firms because of these unexpected hurricanes.

Bowman [15] describes five steps to conduct an event study that we summarize into three main ones:

- Identifying the event of interest and the timing of the event;
- Specifying a benchmark model for normal stock returns behavior; and
- Calculating and analyzing abnormal returns around the event date.

Hurricanes in the East Atlantic Area tend to occur during the summer season. Specifically, the Tropical Prediction Centre of the National Weather Service determines that the hurricane season starts on 1 June and finishes on 30 November within the year. Although sometimes they can be early or late, all the hurricanes selected take place within this period. In our study, hurricane Sandy is situated in the borderline of the season as it lasted until the end of October, whereas the remaining six hurricanes took place mainly at the end of August and September. All of them have common characteristics in terms of magnitude, period, strength and area of landfall. It is important to note that hurricane Sandy is a little bit different for some reasons: the category of this hurricane is lower than the others (Category 3, while others were Categories 4 and 5). Apart from this, it is surprising how a hurricane with a lower category can have produced higher damages than others with stronger category. The explanation of this is that Sandy affected some densely-populated areas such as Washington, DC and New York. The damage comes from the intensity of the storm and from the area affected. Wall Street was even closed for two days due to hurricane Sandy. It was the first closure since the terrorist attacks in September 2001.

As suggested by Ewing et al. [5], the landfall of the hurricane is set as the event date in our study, which is denoted as *t* = 0. 

The timeline is divided into three temporary windows: the estimation window, the event window and the post-event window.

- Estimation window: It is the period between *T*_0_ and *T*_1_. This period comprises 150 trading days. In this period, the *Market model* is applied for estimating normal returns.
- Event window: This period ranges from *T*_1_ to *T*_2_ and *t* = 0 (the hurricane landfall) is situated in the middle of this period. This is composed of twenty-one trading days: ten before the event date and ten after the event date.
- Post-event window: which comprises the period from *T*_2_ to *T*_3_. This period will be used to prove and check if the daily returns of the companies selected go back to the previous situation before the hurricane.

Depending on the length of the event window, we can differentiate between short-horizon and long-horizon event studies. There is no specific length established for distinction between long and short events. For instance, Kothari and Warner [16] established short-horizon less than 12 months and long-horizon from 12 or more. In our analysis, we conduct a short-horizon event study as it improves the methodology, allowing better accuracy in measuring the effect of the announcement of the event [16].

For the sample of hurricanes, their respective landfalls have been considered as the event date; that is, *t* = 0. However, for the cases of hurricanes Rita and Ike, their landfalls took place at weekends, so, since those days are not tradable, the event date were moved to the next Monday, being the new landfall for hurricane Rita on 26 September and for Ike on 8 September.

Regarding the length of the estimation window, we can find two types of estimation windows and choose the one which best fit our research. A long estimation window is better as it increases the accuracy thanks to all the samples involved on it. However, this increase can produce two hurricanes coexist, affecting the results of the others. For instance, this would happen in our research as Rita and Katrina happened in 2005. Both took place in the hurricane season and with less than one month between them. Despite the lack of consensus about the length of the estimation window, a period of 120–200 trading days is the most appropriate according to the existing literature. Following Lamb [6], the set of dates from which the parameters are estimated includes the 150-trading day period ending ten days prior to 22 September 1989 in the case of Hurricane Hugo and 150-trading day period ending ten days prior to 24 August 1992 for Hurricane Andrew. In this sense, for each of the seven hurricanes analyzed, 150-trading days are used as the estimation window, whereas the event window is set in ten days before and ten days after the event (landfall). The static estimation window deviates from the traditional event study. Nevertheless, it is the optimal one for researches such as this one. Lagged estimation window has been traditionally used, such as the 10 trading days prior to the event study to estimate normal performance. Lagged estimation windows are problematic for this study, because often time in the two-week window before hurricane landfall another storm is hitting the Gulf, e.g., Katrina and Rita Hurricane. A lagged estimation window that covers landfall of a previous hurricane will generate a significantly inaccurate beta, and the bias in this beta will impact the calculation of expected and abnormal returns affecting the final decisions. A static estimation window assures that beta is free of bias, and if we assume that individual hurricanes themselves do not impact in individual firm’s sensitivity to market returns there is no need for a lagged estimation window anyway [11].

Conceptually, event study makes a difference between two cases: the expected events which show the situation without any event would have taken place and returns emerged from an unexpected event, that is, abnormal returns.

Abnormal return (AR) is defined as the difference between the current return of the stock and the normal or expected return in case the event did not take place.

(1) $$AR_{it}=R_{it}-NR_{it}$$

To determine the normal return, we use the estimation window we have previously defined. This estimation window comprises the period between T_0_ and T_1_ and is composed of 150 trading days. This period, usually designed as benchmark, is necessary to study the normal behavior of the stock prices before the event date. 

From the three kinds of the existing techniques (mean adjusted, market, and market-adjusted model), proposed by Peterson [17], the most appropriate for our research is the market model to estimate the expected return [18,19,20,21]. The Market model [22] adjusts for the stock’s systematic risks in estimating the stock abnormal return. In this way, the variance of the abnormal return will be reduced because one is removing the portion of the return that is related to the market index [23]. The market model relates the return of any stock to the return of the market portfolio:

(2) $$R_{it}=\alpha_{i}+\beta_{i}R_{mt}+\varepsilon_{it}$$

where *R_it_* is the return of the stock *i* on Day “*t*”; *R_mt_* is the return of the market portfolio on Day “*t*”; α_i_ is the constant term; β_i_ is a measure of the sensitivity between *R_it_* with respect to *R_mt_*; and $\varepsilon_{\mathrm{it}}$ is the random disturbance term.

In our study, *R_it_* is the daily return P & C Company, and *R_mt_* is the daily return of the S & P 500. The parameters α and β are estimated by Ordinary Least Squares (OLS):

(3) $$NR_{it}=\hat{\alpha_{i}}+\hat{\beta_{i}}R_{mt}$$

where $NR_{it}$ is the normal return of one of our stocks “*i*” is at time “*t*”. *R_mt_* is the daily market index (S & P 500) return. $\hat{\alpha_{i}}$ and $\hat{\beta_{i}}$ are OLS estimates of the regression coefficients. Since stocks returns can exhibit autorregressive conditional heteroscedasticity, we have computed the quasi-maximum likelihood covariances and standard errors as described in [24]. The model is estimated under the assumption that the errors are conditionally normally distributed.

From the previous two equations, we can interpret the abnormal returns as the residuals of the benchmark model. We now assume we have seven firms in the sample (N = 7) and we define a matrix of abnormal returns (AR) by adding each abnormal return of each hurricane to the matrix as follows:

(4) $$AR=\left(\begin{array}{ccc} AR_{1,T_{1}} & \ldots & AR_{N,T_{1}}\\ \vdots & & \vdots \\ {AR}_{1,0} & \ldots & {AR}_{N,0}\\ \vdots & & \vdots \\ {AR}_{1,T_{2}} & \ldots & {AR}_{N,T_{2}} \end{array}\right)$$

The column of the matrix is a time series of abnormal returns for firm “*i*” in which the time index “*t*” is counted from the event date. Each row corresponds to the cross section of abnormal returns for each time point in the event window. To analyze how the reactions of our stocks are around the hurricane landfalls, we should address separately each firm’s return series.

However, this does not contribute much as some of the movements of the stock prices are not only caused by the hurricanes but also by other information which is not related to the event we are currently examining. Thus, we use the unweight cross-sectional average of abnormal returns (AAR) in period *t*, which is set as follows:

(5) $$AAR=\bar{AR_{it}}=\frac{1}{N}\overset{N}{\underset{i=1}{\sum }}AR_{it}$$

Moreover, it is also interesting to study cumulative abnormal returns within the event window, which are calculated by the aggregation of $AR_{it}$ from $T_{1}$ to $T_{2}$:

(6) $$CAR_{i}=\overset{T_{2}}{\underset{t=T_{1}}{\sum }}AR_{it}$$

Finally, in event studies, $CAR_{i}$ is usually aggregated over the cross-section, resulting in cumulative average abnormal returns (CAAR):

(7) $$CAAR=\bar{CAR}=\frac{1}{N}\overset{N}{\underset{i=1}{\sum }}CAR_{i}$$

$\bar{CAR}$ can also be obtained by aggregating $\bar{AR}$ values over time:

(8) $$\bar{CAR}=\overset{t_{2}}{\underset{t=t_{1}}{\sum }}{\bar{AR}}_{t}$$

In summary, in event studies, abnormal returns are calculated as an outcome, by averaging ($\bar{AR}$), cumulating over time (CAR), and averaging again ($\bar{CAR}$). The last step in event study methodology is to test the statistical significance of results by verifying that changes in stock prices are not random. 

To test the significance of cumulative abnormal returns, we first apply the parametric *t*-test. The traditional *t*-test relies on the assumption that the abnormal returns are normally distributed 

(9) $$AR_{t}\sim N\left(0,\frac{\sigma^{2}}{N}\right)$$

As the variance $\sigma^{2}$ is not known, the variance of the residuals obtained in the estimation period of the regression model is used as an estimator [18]. The null hypothesis is that stock prices do not respond to the event. If the abnormal returns are independent and identically distributed, the statistic follows a Student’s t-distribution under this hypothesis. For the average abnormal returns, the statistic is defined as:

(10) $$t=\frac{\bar{AR_{t}}}{\sigma \left(AR_{t}\right)}$$

Similarly, for the accumulated abnormal returns, the statistic is:

(11) $$t=\frac{CAR_{T_{1},T_{2}}}{\sigma \left(CAR_{T_{1},T_{2}}\right)}$$

The null hypothesis is that the expected cumulative return is equal to zero. Furthermore, we also carried out two nonparametric tests. According to Campbell and Wasley [12], the inclusion of nonparametric test checks the robustness of the conclusions based on the parametric test. In this particular case, we used the sign test [23] and the Wilcoxon test [25]. The sign test is a binomial test, which calibrates if the frequency of abnormal positive residuals is equal to 50%. To implement this test, we should determine the proportion of values in the sample that shed no negative abnormal returns under the null hypothesis. The null value is calculated as the average fraction of stocks with no negative abnormal returns in the estimation period. If abnormal returns are independent, under the null hypothesis, the number of positive abnormal return values follows a binomial distribution with parameter *p*, and the statistic, *z*, is:

(12) $$z=\frac{|p_{0}-p|}{\sqrt{p(1-p)N}}$$

where *p*_0_ reflects the observed proportion of positive returns for a given time window. This statistic is distributed as a normal law of variance 1 and mean 0.

The Wilcoxon test considers both the sign and the magnitude of abnormal returns, with the statistic:

(13) $$W=\sum_{i=1}^{N}r_{i}^{+}$$

where, $r_{i}^{+}$ is the positive range of the absolute value of abnormal returns. This test assumes that none of the absolute values are the same and each is non-zero. Under the null hypothesis that the probability of positive and negative abnormal returns is equal, when *N* is large, *W* asymptotically follows a normal distribution with a mean *E(W)* and variance *V(W)* as follows, respectively:

(14) $$E(W)=\frac{N(N+1)}{4}$$

(15) $$V(W)=\frac{N(N+1)(2N+1)}{12}$$

## 4. Research Design 

In our study, we run a standard short-horizon event study. Thus, we first need to estimate the abnormal return for each P & C Insurance Company relative to the stock market. Secondly, we calculate the average abnormal returns for each hurricane analyzed, as well as the cumulative average abnormal returns for the whole sample. If those cumulative abnormal returns are statistically significant in the selected event window, then the weak form of the market efficiency does not hold. In total, we have 49 observations, resulting from seven U.S. P & C Insurance Companies and seven major hurricanes that hit the east coast of the U.S. from 2005 to 2012. We do not know beforehand if hurricanes could affect in a negative or in a positive way, but according to existing literature both effects on returns are possible. We could also find hurricanes did not produce any abnormal return on the companies chosen. In this sense, Lamb [6] argue that despite the heavy damage produced by Hugo in 1989, the hurricane was considered a non-event in terms of a significant price reaction.

Furthermore, for our research we have conducted an event window composed of ten trading days before and ten trading days after the event. This is designed as follows: E (−10, +10). Note that the event day does not belong to any of the ten days, that is, it is independent of them. Our event window will consist of twenty-one days.

The election of this event window is the result of a study among all the hurricanes selected for this research. When a tropical storm transforms into a hurricane, it passes some days since the storm reaches its maximum category and until the hurricane reaches landfalls. This is not a regular or a fixed number of days and it varies from one hurricane to other. Moreover, once the hurricane has made landfall, it passes another period of days till this lose part of its strength as it converted again in a residual storm which tends to disappear. If we selected an event window with fewer days, this would suppose some hurricanes were left outside the event window chosen even when they were still considered as hurricanes. With ten days before and after the event day, we assure all the hurricanes are framed inside since they reach the category to be considered a hurricane until it tends to disappear.

The next step is to calculate both the daily P & C Companies stock and S & P 500 returns. In our case, we set as the market benchmark. This is computed for both the selected as well as for the market index. To compute the returns, we apply the following formula applying natural logarithm [26]. Once the returns have been obtained, it is time to estimate the expected returns by using the Ordinary Least Squares (see Equation (2)). The way to calculate the stock’s abnormal returns (*AR_it_*) is by subtracting the current return minus the expected return (see Equation (1)). Then, to compute the global sample, we create a matrix, *AR*, composed of the abnormal returns of the P & C Insurance Companies for the event window E (−10, +10). The informativeness of the analysis is greatly improved by averaging the information over the sampled firms so the unweighted cross-sectional average of abnormal returns is considered [17]. Thus, we compute the average abnormal return (see Equation (5)). Reaching this point, we conduct a normality test to check whether the sample follow a normal distribution or not. Anderson–Darling (A-D) or Kolmogorov–Smirnov (K-S) tests can be suitable for such analysis. According to Engmann [27] the A-D test requires less data than the Kolmogorov Smirnov test to reach sufficient statistical power. They state that the A-D test is more sensitive to the tails of distributions and it is more reliable than the K-S. Consequently, we have conducted the Anderson–Darling test on the average abnormal return (AAR) of our event window. 

**Figure 1:**
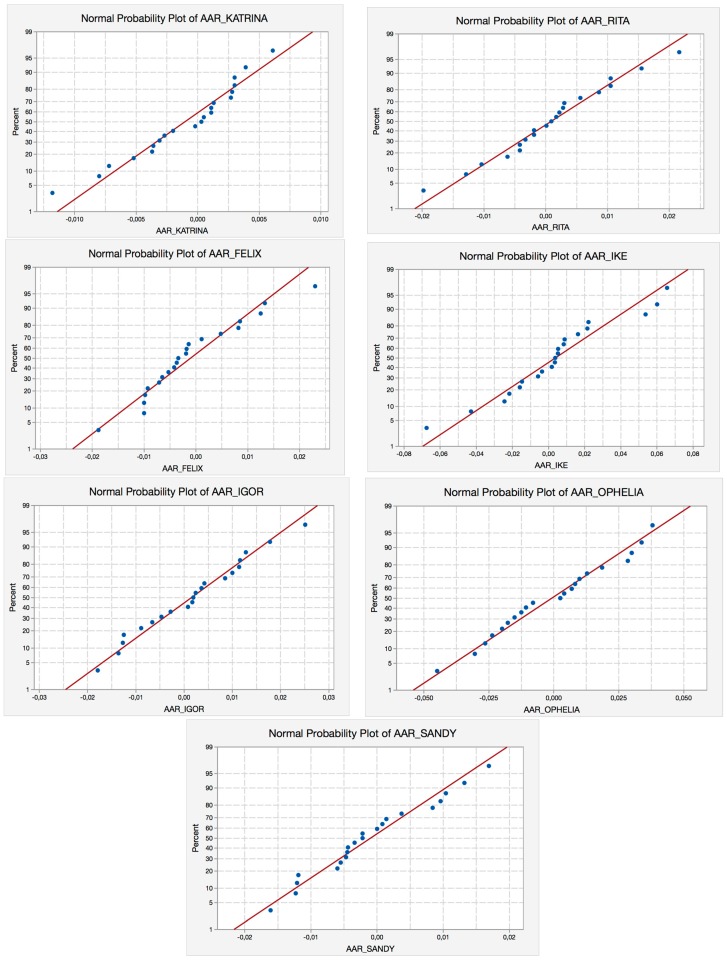
P-P plots for hurricanes selected for E (−10, +10). Source: Self-Compilation.

As we can observe in Table 5, the Anderson-Darling (AD) test does not reject any of the hurricanes. We also present (Figure 1) the corresponding P-P Plots of the seven hurricanes for E (−10, +10).

The next step would be to test the significance of the Cumulative Average Abnormal Returns (CAAR). The first step here is to test our first hypothesis, that is, whether the mean of the CAAR is equal or different to zero, which is defined as follows using a parametric test (*t*-test).

(16) $$H_{0}=E\left(CAAR_{it}\right)=0\;H_{1}=E\left(CAAR_{it}\right)\neq 0$$

We test the hypothesis using a bootstrapped estimation, as otherwise it would be costly and long. This process it is done repetitively from the sample of cumulative abnormal return for each hurricane [11].

The statistical reaction of our sample will allow accepting or rejecting the null hypothesis and determine if the U.S. Stock Market of the United States reacted in one or other way to the hurricanes selected as it has been defined by the EMH. As Hewitt [11] estates in his research, “this finding is important, as an investor with this knowledge does not need to know the characteristics of a hurricane or the macro environment that cause inefficiency in individual hurricanes to make profit, he or she only needs to execute their strategy over all hurricanes”.

Moreover, to test the previous hypotheses, we also carry out some non-parametric tests apart from the parametric one used before. Non-parametric tests are used when the data to study do not meet the requirement for parametric tests. Luoma [28] states that a non-parametric test makes no hypothesis about the value of a parameter in a statistical density function. Accordingly, to the author, the non-parametric tests are going to be used for testing cumulative average abnormal returns (CAARs). Thus, we have used the Sign test as well as the Wilcoxon test.

## 5. Findings and Results

As mentioned in the previous section, a parametric test (*t*-test) is first conducted to study the reactions of the hurricanes selected in the P & C Insurance Companies. It must be proved if the expected CAARs of each hurricane are equal or different from 0 for the event window E (−10, +10). Being different from 0 indicates the existence of an abnormal behavior due to the hurricanes selected. In terms of statistics, it will mean the rejection of $H_{0}$ and, consequently, the acceptance of $H_{1}$. In other words, assuming a specific confidence level (i.e., 95%), if *p*-value is less than or equal to the significance level (0.05), the decision is to reject the null hypothesis and conclude that the mean of CAARs and the hypothetical mean (zero) is statistically different. On the contrary, if *p*-value is greater than the significance level (0.05), the decision fails to reject the null hypothesis.

In Table 6, the information concerning the *t*-test is summarized for all the hurricanes. 

**Figure 2:**
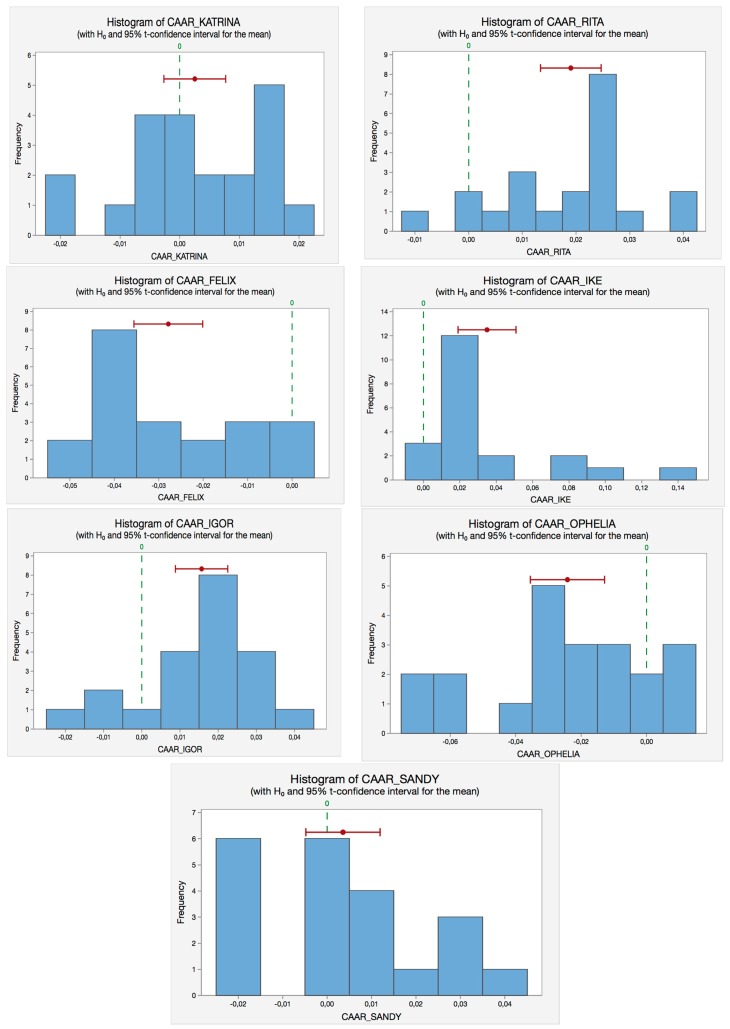
*t*-Test histograms for the hurricanes selected. Source: Self-Compilation.

From Table 6, and applying the rule of thumb, we accept $H_{0}$ if the *p*-value is greater than the α-level, as in the case of Hurricane Katrina and Sandy. On the other hand, for the rest of the hurricanes (Rita, Felix, Ike, Igor and Ophelia), their *p*-values are smaller than 0.05, rejecting the null hypothesis. In such hurricanes, we find significant cumulative abnormal returns during the event window, E (−10, +10). Figure 2 illustrates the corresponding histograms of the *t*-tests for each hurricane to show the same results in a more intuitive and visual way; the green dot line represents the null hypothesis value (equal to zero) whereas the red line illustrates the observed value.

Although our sample is assumed to follow a normal distribution, apart from applying this parametric test, we have also carried out two non-parametric tests in order to reinforce our research, Sign Test and Wilcoxon Test. Table 7 summarizes the results of the Sign test for all the hurricanes.

The sign test does not always achieve the specified confidence level (i.e., 95%) because the statistic is discrete. That is why three confidence intervals with varying levels of precision are provided in Table 7. For example, in the case of Katrina, the median for CAARs is 0.0009 and we are 95% confident that it will be in the interval (−0.0027979; 0.0118795). To test whether the difference between the median and the hypothesized median is statistically significant, we compare the *p*-value to the significance level (0.05). Since the *p*-value is higher than 0.05, we fail to reject the null hypothesis, that is, we cannot conclude that the median of CAARs differs from the hypothetical one.

**Figure 3:**
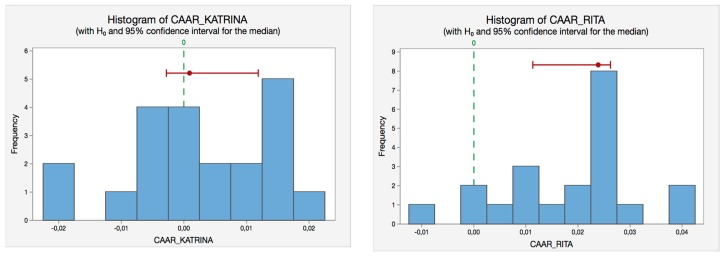

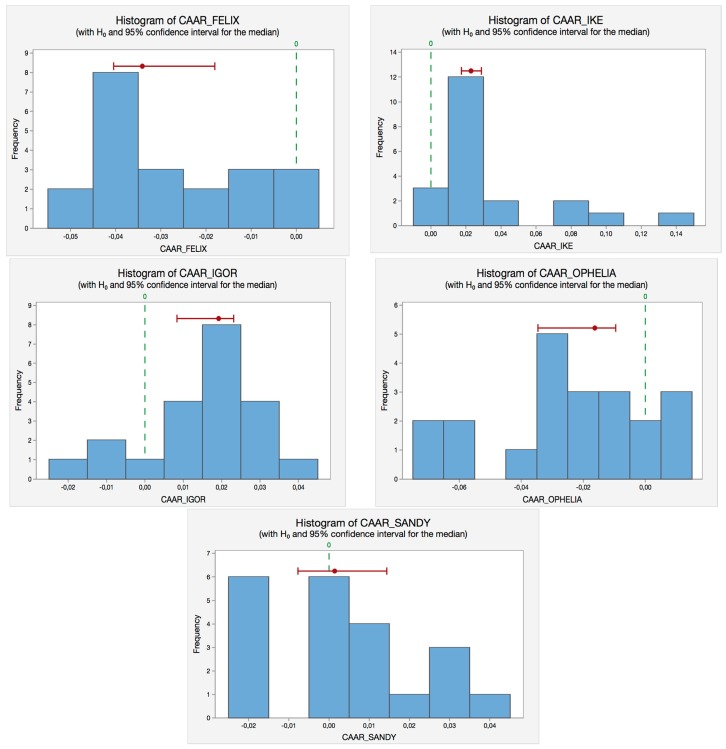
Sign-test histograms for the hurricanes selected. Source: Self-Compilation.

We also include the corresponding histograms (see Figure 3) of the Sign-tests for each hurricane to illustrate the same reasoning in a visual way.

The second non-parametric test we carry out is the Wilcoxon test whose results are summarized in Table 8.

As in the previous test, the specified confidence level (i.e., 95%) is not always achieved because the Wilcoxon statistic is discrete. Thus, we use a normal approximation with a continuity correction to estimate the closest achievable confidence level. For example, in the case of Katrina, the median for CAARs is 0.00355 and we are 94.84% confident that it will be in the interval (−0.00275; 0.00750). To test whether the difference between the median and the hypothesized median is statistically significant, we compare the *p*-value to the significance level (0.05). Since the *p*-value (0.3662) is higher than 0.05, we fail to reject the null hypothesis; that is, we cannot conclude that the median of CAARs differs from the hypothetical one. 

**Figure 4:**
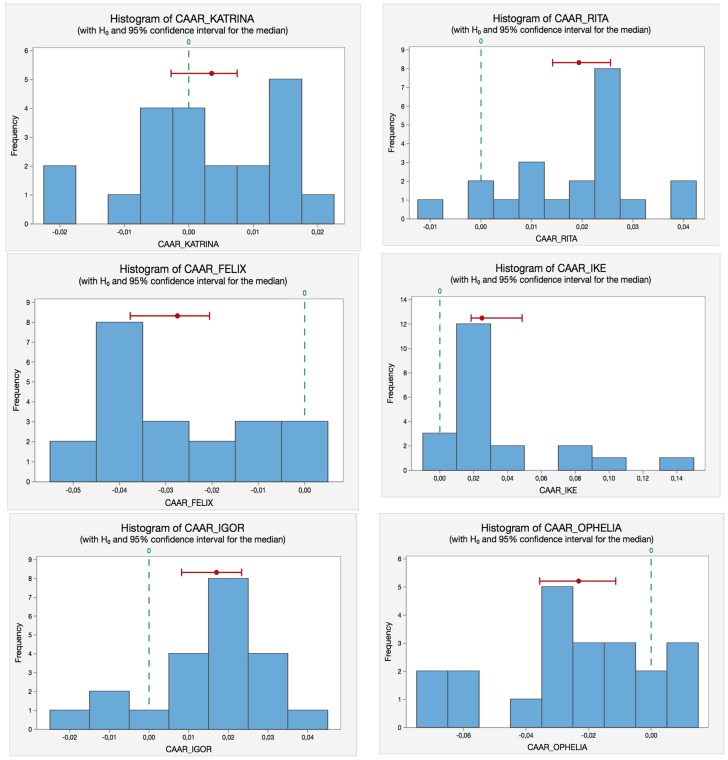

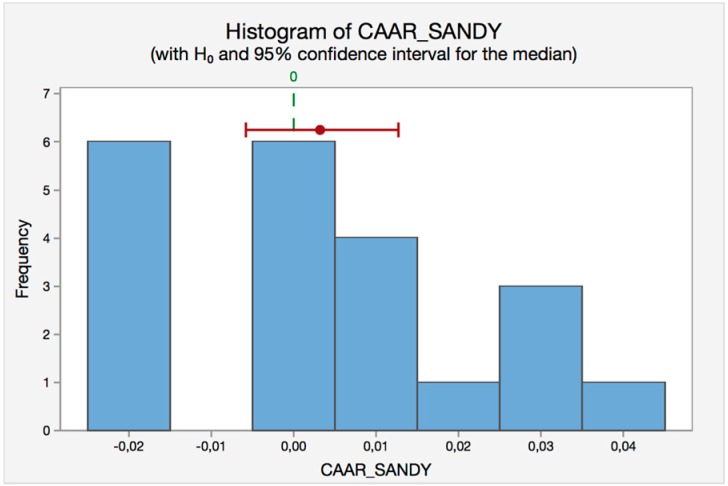
Wilcoxon-test histograms for the hurricanes selected. Source: Self-Compilation.

We also include the corresponding histograms (see Figure 4) of the Wilcoxon-test for each hurricane to illustrate the same reasoning in a visual way. Notice that both non-parametric tests, Sign test (Figure 3) and Wilcoxon test (Figure 4), provide similar results.

## 6. Conclusions

In this study, the financial impact of the most recent hurricanes to hit the U.S. East Coast on the main U.S. P & C Companies, listed in the NYSE, are analyzed. To conduct this research, a standard short horizon event study technique around the landfall of such hurricanes has been used. 

In total, 49 event studies were conducted on seven major hurricanes from 2005 to 2012; all of them selected depending on some key characteristics. This information has been carefully collected from the official Website of National Hurricane Center and then structured in terms of wind speed, area affected, category, direction, etc. This is essential to ensure the good understanding of the research as well as the minimization of possible bias from non-official information sources.

In general, we find different reactions in the U.S. market depending on the hurricane selected. For most of the hurricanes analyzed (Rita (2005), Felix (2007), Ike (2008), Igor (2010) and Ophelia (2012)), we find evidence of the significant impact on the insurance stock returns indicating that, in the very short term around the hurricane strikes, the expected cumulative abnormal returns are significantly different from zero; in other words, demonstrating that the Efficient Market Theory (EMT) does not hold. Such results are obtained from conducting both parametric and non-parametric statistical tests.

On the contrary, the sample of P & C Insurance Firms seems insensitive to both Hurricane Katrina (2005) and Hurricane Sandy (2012) in terms of cumulative average abnormal returns from 10 days before to 10 days after the landfall. Our findings are aligned with Merill Lynch and Baker [29,30] about Hurricane Katrina, highlighting that the short-term economic impact was small and the market’s resilience in the days following the storm appeared to indicate that investors did not panic and did not overreact to the short-term developments. On the other hand, the forecast for Hurricane Sandy’s storm track proved to be accurate, with the European Centre for Medium-Range Weather Forecasts (ECMWF) providing the track accurately more than a week in advance. This gave adequate time for a set of immediate preparations to be instigated. 

## Figures and Tables

**Table 1:** Main hurricanes.

Name	Event Date *	Category (By Saffir-Simpson Hurricane Wind Scale (SSHWS))	Damage (in USD)
Katrina	23–31 August 2005	5	148 Billion
Rita	18–26 September 2005	5	12.037 Billion
Felix	31 August–5 September 2007	5	850 Million
Ike	1–14 September 2008	4	29.5 Billion
Igor	8–23 September 2010	4	25 Billion
Ophelia	20 September–3 Octorber 2011	4	21 Billion
Sandy	22–29 Octorber 2012	3	71 Billion

Source: Self- Compilation based on the National Hurricane Centre (NHC); * Event date corresponds to the period since the hurricane is considered a hurricane due to its strength until it loses this strength and tends to disappear. Within this period, the landfall takes place.

**Table 2:** Sample of P & C Companies.

Ticker	Company	Total Revenues * (in USD)
MMC	Marsh & McLennan Companies	12,261 Billion
PGR	Progressive Corporation	18,170 Billion
ACE	Ace Limited	19,261 Million
ALL	The Allstate Corporation	34,507 Million
CB	The Chubb Corporation	13,502 Million
TRV	Travelers	26,191 Million
BRK-B	Berkshire Hathaway’s	182,150 Million

Source: Self-Compilation based on NYSE; * at 31 December 2013.

**Table 3:** Categories of hurricanes.

Category	Wind Speed	Characteristics
1	119–153 km/h	Very dangerous winds. Extensive damage to power lines and poles. Large branches of trees will snap and shallowly rooted trees may be toppled.
2	154–177 km/h	Extremely dangerous winds. Well-constructed frame houses could suffer damages in roof and siding damages.
3 (major)	178–208 km/h	Devastating damage will occur. No water or electricity services available. Houses will suffer damage or removal of roof docking and gable ends. Trees will be uprooted.
4 (major)	209–251 km/h	Catastrophic events will occur. Damages on roof structures and some exterior walls. Trees and power poles downs. Power outages for weeks to months. The area will be uninhabitable for weeks or months.
5 (major)	252 km/h or more	Catastrophic damage will occur. High percentage of homes destroyed. Isolation of residential areas due to fallen trees and power poles. Area uninhabitable.

Source: National Hurricane Center (NHC).

**Table 4:** Main areas affected by the hurricanes.

Hurricane	Main Areas Affected
Katrina	New Orleans and Mississippi coast
Rita	Texas, Louisiana and Florida Keys
Felix	Netherlands Antilles and Nicaragua
Ike	Caribbean, Texas and Louisiana
Igor	Bermuda and Newfoundland
Ophelia	Bermuda and Leward Island
Sandy	Jamaica, Cuba and Bahamas

Source: Self-Compilation.

**Table 5:** Anderson-Darling (A-D) test on average abnormal returns.

**Hurricane Katrina**					
Sample Size	21				
Statistics	0.41432				
Rank	14				
Α	0.2	0.1	0.05	0.02	0.01
Critical value	1.3749	1.9286	2.5018	3.2892	3.9074
Reject	No	No	No	No	No
**Hurricane Rita**					
Sample Size	21				
Statistics	0.21532				
Rank	16				
Α	0.2	0.1	0.05	0.02	0.01
Critical value	1.3749	1.9286	2.5018	3.2892	3.9074
Reject	No	No	No	No	No
**Hurricane Felix**					
Sample Size	21				
Statistics	0.52823				
Rank	22				
Α	0.2	0.1	0.05	0.02	0.01
Critical value	1.3749	1.9286	2.5018	3.2892	3.9074
Reject	No	No	No	No	No
**Hurricane Ike**					
Sample Size	21				
Statistics	0.5101				
Rank	19				
α	0.2	0.1	0.05	0.02	0.01
Critical value	1.3749	1.9286	2.5018	3.2892	3.9074
Reject	No	No	No	No	No
**Hurricane Igor**					
Sample Size	21				
Statistics	0.18979				
Rank	5				
α	0.2	0.1	0.05	0.02	0.01
Critical value	1.3749	1.9286	2.5018	3.2892	3.9074
Reject	No	No	No	No	No
**Hurricane Ophelia**					
Sample Size	21				
Statistics	0.22014				
Rank	6				
α	0.2	0.1	0.05	0.02	0.01
Critical value	1.3749	1.9286	2.5018	3.2892	3.9074
Reject	No	No	No	No	No
**Hurricane Sandy**					
Sample Size	21				
Statistics	0.34723				
Rank	15				
α	0.2	0.1	0.05	0.02	0.01
Critical value	1.3749	1.9286	2.5018	3.2892	3.9074
Reject	No	No	No	No	No

Source: Self-Compilation.

**Table 6:** *t*-Tests on CARs for hurricanes selected.

Hurricane	N	Mean	StDev	St Error Mean	95% CI for the Mean	*t*-Value	*p*-Value
Katrina	21	0.002529	0.011378	0.002483	(−0.002651; 0.007708)	1.02	0.3207
Rita	21	0.019033	0.012403	0.002707	(0.013388; 0.024679)	7.03	<0.0001 *
Felix	21	−0.027852	0.017014	0.003713	(−0.035597; −0.020108)	−7.5	<0.0001 *
Ike	21	0.034971	0.034986	0.007634	(0.019046; 0.050897)	4.58	0.0002 *
Igor	21	0.015657	0.015056	0.003285	(0.008804; 0.022510)	4.77	0.0001 *
Ophelia	21	−0.02419	0.024902	0.005434	(−0.035526; −0.012855)	−4.45	0.0002 *
Sandy	21	0.003562	0.018347	0.004004	(−0.004790; 0.011913)	0.89	0.3842

Source: Self-Compilation; * *p*-value < 0.05.

**Table 7:** Sign-test on CAARs for the hurricanes selected.

Hurricane	N	Median	95% CI for the Median	Achieved Confidence	Position	*p*-Value
Katrina	21	0.0009	(−0.0027000; 0.0109000)	92.16%	(7; 15)	1
			(−0.0027979; 0.0118795)	95.00%	Interpolation	
			(−0.0030000; 0.0139000)	97.34%	(6; 16)	
Rita	21	0.0239	(0.0116000; 0.0262000)	92.16%	(7; 15)	<0.0001
			(0.0113388; 0.0262653)	95.00%	Interpolation	
			(0.0108000; 0.0264000)	97.34%	(6; 16)	
Felix	21	−0.0341	(−0.0400000; −0.0219000)	92.16%	(7; 15)	<0.0001
			(−0.0404571; −0.0180474)	95.00%	Interpolation	
			(−0.0414000; −0.0101000)	97.34%	(6; 16)	
Ike	21	0.0229	(0.0182000; 0.0278000)	92.16%	(7; 15)	<0.0001
			(0.0174491; 0.0288448)	95.00%	Interpolation	
			(0.0159000; 0.0310000)	97.34%	(6; 16)	
Igor	21	0.0192	(0.0089000; 0.0225000)	92.16%	(7; 15)	0.0072
			(0.0083450; 0.0231203)	95.00%	Interpolation	
			(0.0072000; 0.0244000)	97.34%	(6; 16)	
Ophelia	21	−0.0163	(−0.0345000; −0.0111000)	92.16%	(7; 15)	0.0072
			(−0.0346306; −0.0095655)	95.00%	Interpolation	
			(−0.0349000; −0.0064000)	97.34%	(6; 16)	
Sandy	21	0.0014	(−0.0041000; 0.0142000)	92.16%	(7; 15)	1
			(−0.0077240; 0.0143632)	95.00%	Interpolation	
			(−0.0152000; 0.0147000)	97.34%	(6; 16)	

Source: Self-Compilation.

**Table 8:** Wilcoxon Test on CAARs.

Hurricane	N	Median	95% CI for the Median	Achieved Confidence	Wilcoxon Statistic	*p*-Value
Katrina	21	0.00355	(−0.00275; 0.00750)	94.84%	142	0.3662
Rita	21	0.01935	(0.01415; 0.02560)	94.84%	227	0.0001
Felix	21	−0.0275	(−0.03770; −0.02055)	94.84%	2.5	<0.0001
Ike	21	0.0249	(0.0185; 0.0486)	94.84%	231	<0.0001
Igor	21	0.017	(0.00820; 0.02335)	94.84%	214	0.0007
Ophelia	21	−0.0232	(−0.03565; −0.01135)	94.84%	16	0.0006
Sandy	21	0.0032	(−0.0058; 0.0127)	94.84%	132.5	0.5663

Source: Self-Compilation.
